# Biomarkers of clinical benefit for anti-epidermal growth factor receptor agents in patients with non-small-cell lung cancer

**DOI:** 10.1038/bjc.2011.207

**Published:** 2011-06-07

**Authors:** A G Pallis, D A Fennell, E Szutowicz, N B Leighl, L Greillier, R Dziadziuszko

**Affiliations:** 1 Department of Medical Oncology, University General Hospital of Heraklion, PO Box 1352, Heraklion 71110, Crete, Greece; 2 Queen's University Belfast, Centre for Cancer Research and Cell Biology & Northern Ireland Cancer Centre, 97 Lisburn Road Belfast, BT9 7BL Northern Ireland, UK; 3 Department of Oncology and Radiotherapy, Medical University of Gdansk, 7 Debinki Street, 80-211 Gdansk, Poland; 4 Department of Medical Oncology, Princess Margaret Hospital, University of Toronto, Toronto, Ontario, Canada; 5 Department of Thoracic Oncology, Assistance Publique–Hôpitaux de Marseille, Faculté de Médecine, Université de la Méditerranée, Marseille, France

**Keywords:** EGFR, immunohistochemistry, gene copy number, mutations, gefitinib, erlotinib

## Abstract

Non-small-cell lung cancer (NSCLC) remains by far the major cause of cancer-related death in the Western world in both men and women. The majority of patients will be diagnosed with metastatic disease, and chemotherapy doublets remain the cornerstone of treatment for these patients. However, chemotherapy has a minimal impact on long-term survival and prognosis remains poor for these patients. Further improvement in treatment is likely to require incorporation of novel targeted therapies. Among these agents, inhibitors of the epidermal growth factor receptor (EGFR) have demonstrated significant activity in the first-, second- or third-line treatment of NSCLC. The purpose of current paper is to present the evidence for using several proposed molecular biomarkers as a tool for selection of NSCLC patients for anti-EGFR treatment. According to current data, *EGFR* mutation status appears to be the strongest predictor for the selection of NSCLC patients to first-line treatment with EGFR tyrosine kinase inhibitors *vs* chemotherapy. Use of other biomarkers remains investigational.

Chemotherapy has been the backbone of treatment for patients with advanced non-small-cell lung cancer (NSCLC) for the last decades; however, it has clearly reached a plateau of activity, and thus further improvements will require integration of novel therapies. Among the targeted agents, epidermal growth factor receptor (EGFR) inhibitors gefitinib and erlotinib are now established as an option for first-, second- or third-line treatment (Shepherd *et al*, 2005; Kim *et al*, 2008; Mok *et al*, 2009; Maemondo *et al*, 2010; Mitsudomi *et al*, 2010) or as maintenance treatment (Cappuzzo *et al*, 2010a). Furthermore, the addition of cetuximab, a monoclonal antibody, against the extracellular domain of EGFR to the vinorelbine/cisplatin doublet resulted in a statistically significant, but modest, survival prolongation (Pirker *et al*, 2009).

A subset of patients treated with EGFR inhibitors experience a clinical benefit and even these patients eventually develop disease progression. It is clear that we need to identify reliable predictive factors that will allow for the selection of patients who are most likely to benefit from a particular agent, while sparing others from toxicity of ineffective treatments and the health-care systems from the significant costs of these newer agents. The purpose of the present paper is to focus on the current evidence for using several proposed molecular biomarkers as a tool for selection of NSCLC patients for anti-EGFR treatment.

## Search strategy and selection criteria

A bibliographic search of the Medline database was conducted for papers published from 1 January 2000 to 1 July 2010, with the keywords ‘non-small-cell lung cancer’, ‘epidermal growth factor receptor’, ‘erlotinib’ ‘gefitinib’ and ‘cetuximab’. The search was limited to articles written in English. When considering chemotherapy, targeted therapy or multimodality treatment, only data from phase III trials or randomised phase II trials were incorporated. The Medline search was supplemented by a manual search of meeting abstracts (World Conference on Lung Cancer, European Society of Medical Oncology Annual Congress, American Society of Clinical Oncology Annual Meeting, European Lung Cancer Conference) as well as reference lists of original and review articles. A consensus was reached among all authors for the manuscript.

## Positive predictive factors

### Protein expression by immunohistochemistry

Association of positive EGFR immunostaining, as determined by immunohistochemistry (IHC) in NSCLC specimens, with patient sensitivity to EGFR TKI treatment has been studied extensively with both positive (Cappuzzo *et al*, 2005; Hirsch *et al*, 2007) and negative (Parra *et al*, 2004) results reported. Four placebo-controlled phase III trials have evaluated EGFR TKIs as maintenance (Takeda *et al*, 2010; Cappuzzo *et al*, 2010a; Sequential Tarceva in Unresectable NSCLC (SATURN) and West Japan Thoracic Oncology Group (WJTOG) 0203 trials), second- or third-line treatment (Shepherd *et al*, 2005; Thatcher *et al*, 2005; NCIC Clinical Trials Group BR.21 and Iressa Survival Evaluation in Lung Cancer (ISEL) trials). Another phase III trial, the ATLAS trial (Kabbinavar *et al*, 2010), was designed to evaluate the addition of erlotinib to bevacizumab maintenance in NSCLC patients who have not progressed after first-line chemotherapy plus bevacizumab. Patients with EGFR-expressing tumours had significantly higher response rate (RR) in the BR.21 (*P*=0.03) and ISEL trials (8.2 *vs* 1.5% *P* not reported; Tsao *et al*, 2005; Hirsch *et al*, 2006). In three trials (SATURN, BR.21 and ISEL), patients with tumours showing positive EGFR immunostaining had a significantly reduced risk of death or progression with TKI treatment *vs* placebo (Tsao *et al*, 2005; Hirsch *et al*, 2006; Brugger *et al*, 2009) with hazard ratios (HRs) of 0.68–0.77 in favour of EGFR TKI therapy (Table 1). However, it should be noted that in the ISEL trial, the benefit was of borderline significance (treatment by biomarker interaction test *P*=0.049; Hirsch *et al*, 2006). The WJTOG 0203 was a relatively small and negative trial and the lack of any biomarker published data limits the interpretation and applicability of the findings of this study. In the ATLAS trial, EGFR IHC analysis had no predictive value for progression-free survival (PFS) (Johnson *et al*, 2009). The cut-off point analyses of two large placebo-controlled trials in the second- and third-line setting revealed that the originally proposed criterion to define EGFR positivity (10% of cells with any staining intensity) had the best predictive discrimination (Hirsch *et al*, 2008).

Two phase III trials that compared TKIs with chemotherapy either in first-line (Mok *et al*, 2009) or second-line setting (Kim *et al*, 2008) reported biomarker data with tumour EGFR immunostaining. The Iressa Pan-Asian Study (IPASS) study randomly assigned Asian chemo-naive NSCLC patients (never-smokers or former light smokers with adenocarcinoma) to gefitinib or to paclitaxel/carboplatin chemotherapy (Mok *et al*, 2009). This trial met its primary end point of showing non-inferiority of gefitinib, but furthermore demonstrated its superiority compared with chemotherapy for PFS (HR 0.74, *P*<0.001). The Iressa NSCLC Trial Evaluating Response and Survival *versus* Taxotere (INTEREST) trial was a non-inferiority phase III trial that compared gefitinib with docetaxel as second-line treatment (Kim *et al*, 2008). This study also confirmed that gefitinib was non-inferior to docetaxel in terms of overall survival (OS) (HR 1.020). In these trials, using chemotherapy as the comparator, no predictive value of EGFR IHC analysis was observed for response, PFS or survival, and EGFR protein expression status-by-treatment interaction tests were not significant (Fukuoka *et al*, 2009; Douillard *et al*, 2010).

Two phase III trials have assessed the role of monoclonal anti-EGFR antibody therapy in addition to first-line chemotherapy in the treatment of NSCLC (Pirker *et al*, 2009; Lynch *et al*, 2010). The FLEX trial investigated the combination of cisplatin/vinorelbine plus or minus cetuximab, and demonstrated a statistically significant although modest survival benefit in favour of cetuximab in patients with tumours positive for EGFR protein expression. A second smaller trial, which compared the combination of a taxane/carboplatin plus or minus cetuximab (BMS-099) in unselected patients, failed to show a PFS or survival benefit in favour of the experimental arm. The biomarker analysis did not reveal any association between EGFR protein expression and response, PFS or survival (Khambata-Ford *et al*, 2010).

According to the above studies, EGFR protein positivity is observed in the vast majority of NSCLC tumour specimens (ranging from approximately 70 to 90% in most studies), which makes this marker unlikely to be used in practice for patient selection. Placebo-controlled phase III trials with EGFR TKIs in the second- or third-line setting were the only studies indicating some predictive value of lack of protein expression in selecting patients who do not benefit from these agents, although its predictive discrimination did not meet the expectations of a clinically useful test (i.e., clinically meaningful difference between patient subsets).

### *EGFR* gene copy number

*EGFR* gene copy number, assessed by fluorescence *in situ* hybridisation (FISH), has been tested extensively as a predictive factor for response and survival benefit from TKI treatment. The original classification of FISH positivity includes both gene amplification (rare in NSCLC) and high polysomy (⩾4 copies of the *EGFR* gene in >40% of tumour cell nuclei; Cappuzzo *et al*, 2005). In placebo-controlled studies (BR.21 and ISEL studies; Shepherd *et al*, 2005; Thatcher *et al*, 2005), high *EGFR* copy number was associated with higher response rate and significantly prolonged OS from EGFR TKI treatment (Tsao *et al*, 2005; Zhu *et al*, 2008; Table 2). Moreover, in the BR.21 study, high *EGFR* copy number by FISH was both prognostic for worse survival in untreated patients (*P*=0.025) and predictive of greater survival benefit in erlotinib-treated patients (*P*=0.005). In the ISEL trial, high *EGFR* copy was associated with a survival benefit in patients receiving gefitinib compared with placebo (HR 0.61; *P*=0.067), whereas no benefit was observed in patients with FISH-negative tumours (HR 1.16; *P*=0.417; comparison of HRs high *vs* low copy number; *P*=0.045; Hirsch *et al*, 2006). In patients treated with placebo, high *EGFR* copy was associated with a numerically shorter survival, indicating that copy number might also be prognostic. In the biomarker analysis of the SATURN trial, patients derived a PFS benefit with erlotinib irrespective of *EGFR* FISH status in their tumours (Brugger *et al*, 2009). Similarly, in the biomarker analysis of the ATLAS trial, *EGFR* FISH status had no statistically significant predictive value for PFS, although HRs for PFS were numerically different within patient subsets (Table 2; Johnson *et al*, 2009).

The FISH EGFR assay had no predictive value for survival in randomised trials comparing TKI treatment with chemotherapy (Kim *et al*, 2008; Mok *et al*, 2009). In the INTEREST trial, RR was higher in EGFR FISH-positive patients treated with gefitinib compared with docetaxel (13.0 *vs* 7.4% *P*=0.04; Douillard *et al*, 2010). Overall survival and PFS were similar between the two treatment arms, irrespectively of *EGFR* copy number (OS treatment effect between high and low copy number: HR 1.09 and 0.93, respectively; *EGFR* copy number status-by-treatment interaction test; *P*=0.52). In the IPASS study, EGFR FISH positivity was associated with higher response rate and a borderline PFS benefit from gefitinib when compared with platinum-based chemotherapy (*P*=0.044; Fukuoka *et al*, 2009). Placebo-controlled phase III trials of cetuximab in combination with chemotherapy (FLEX and BMS-099) failed to show an association between *EGFR* gene copy number status and clinical end points, including PFS, OS and RR (O’Byrne *et al*, 2009; Khambata-Ford *et al*, 2010).

A phase II trial was performed with prospective *EGFR* gene copy number assessment (Cappuzzo *et al*, 2007). The trial was not limited exclusively to patients with EGFR FISH positive tumours. The biomarker results indicate that PFS and OS benefit in patients with high *EGFR* gene copy number in their tumours appears to be derived from overlapping *EGFR* mutation positivity.

In summary, *EGFR* copy number is predictive of survival benefit from erlotinib or gefitinib in placebo-controlled trials in patients who failed previous chemotherapy (Tsao *et al*, 2005; Hirsch *et al*, 2006). These observations were not confirmed in clinical trials comparing EGFR TKI treatment with chemotherapy (Kim *et al*, 2008; Mok *et al*, 2009), suggesting that the predictive value of *EGFR* gene copy number assessment is confined to second/third line trials with placebo arm as a comparator. At present, *EGFR* gene copy number testing is not recommended in the selection of first- or second-line treatment of advanced NSCLC patients. Data from phase III trials do not suggest a role for *EGFR* gene copy number in predicting benefit from anti-EGFR monoclonal antibodies in NSCLC.

### Somatic EGFR mutations

Most somatic mutations of the *EGFR* gene observed in NSCLC involve the tyrosine kinase coding domain (exons 18–21). Discovery of these mutations in tumours from NSCLC patients was immediately linked with response to gefitinib (Lynch *et al*, 2004; Paez *et al*, 2004). In placebo-controlled phase III studies of gefitinib (Thatcher *et al*, 2005) and erlotinib (Shepherd *et al*, 2005; Cappuzzo *et al*, 2010a), patients with *EGFR*-mutated tumours had significantly higher RR compared with patients with wild-type tumours. In the BR.21 study, both groups derived a survival benefit (Zhu *et al*, 2008). In the ISEL study, there were too few patients with mutations for survival subset analysis (Hirsch *et al*, 2006), whereas in the SATURN trial, a remarkable PFS benefit was observed in patients with tumours with *EGFR* mutations in the erlotinib arm (HR 0.10; *P*<0.0001; Brugger *et al*, 2009). Similarly, the biomarker analysis of the ATLAS trial reported a significant benefit in terms of PFS in patients with tumours bearing *EGFR* mutations in the erlotinib arm (HR 0.44; Johnson *et al*, 2009).

In the INTEREST trial, *EGFR* mutation-positive patients had significantly longer PFS (HR 0.16; *P*=0.001) and higher RR when treated with gefitinib when compared with docetaxel (ORR 42.1 *vs* 21.1% *P*=0.04; Douillard *et al*, 2010). Patients harbouring *EGFR* mutation-positive tumours had longer survival in both gefitinib and docetaxel groups (median survival 14.2 and 16.6 months, respectively) than in the overall population (7.6 and 8.0 months, respectively), and in the population with wild-type *EGFR* (6.4 and 6.0 months, respectively), indicating that *EGFR* mutations have a positive prognostic role. There was no OS difference between treatment groups according to *EGFR* mutation status (subset of patients with mutated tumours, HR=0.83 *vs* those with wild-type *EGFR*, HR=1.02, interaction test; *P*=0.59; Douillard *et al*, 2010). In the IPASS study, patients with *EGFR-*mutated tumours had significantly higher RR with gefitinib compared with chemotherapy (71.2 *vs* 47.3% *P*=0.0001; Fukuoka *et al*, 2009). There was also a striking difference in PFS in patients with *EGFR-*mutated tumours treated with gefitinib compared with those treated with chemotherapy (9.5 *vs* 6.3 months; HR=0.48; *P*<0.001). The predictive role of *EGFR* mutation was also demonstrated by the noteworthy differences in PFS observed in patients with *EGFR* mutation-positive or -negative tumours when treated with gefitinib (9.5 *vs* 1.5 months). In patients without EGFR TK mutations, PFS was significantly superior in the group treated with chemotherapy compared with gefitinib (HR=2.85; *P*<0.001; Table 3). The results of two phase III Japanese trials comparing gefitinib and chemotherapy as first-line treatment in NSCLC patients exclusively with tumours harbouring *EGFR* mutations confirmed improved outcomes with EGFR TKIs (Maemondo *et al*, 2010; Mitsudomi *et al*, 2010). Similar results were also observed in a Chinese trial with erlotinib (Zhou *et al*, 2010).

The NSCLC cell lines harbouring *EGFR* gene mutations are less sensitive to monoclonal antibodies than to EGFR tyrosine kinase inhibitors (Mukohara *et al*, 2005). In the BMS-099 trial, *EGFR* mutation status did not predict benefit from concurrent treatment with cetuximab and chemotherapy. Survival tended to be longer in patients with mutated *EGFR* compared with those with wild-type *EGFR* (HR 0.61; *P*=0.09). This trend was more apparent in the chemotherapy group (HR 0.46; *P*=0.06) than in the cetuximab group (HR 0.84; *P*=0.66), confirming the prognostic role of *EGFR* mutations (Khambata-Ford *et al*, 2010).

Based on the above mentioned trials, *EGFR* mutation testing is now recommended as part of routine care of NSCLC patients to guide decisions about first-line treatment.

### Germline EGFR polymorphisms

Regulatory sequences of the *EGFR* gene are located within the 5′ flanking region, and a highly polymorphic (CA)_*n*_ repeat is situated in intron 1 of the gene. *In vitro* as well as *in vivo* data indicate that *EGFR* transcriptional activity may be influenced by the number of CA repeats (Gebhardt *et al*, 2000). Given the association between gene expression and the number of CA repeats, the efficacy of anti-EGFR treatment could vary according to a patient's genotypic differences. Two clinical single cohort studies in Asian patients (Han *et al*, 2007; Nie *et al*, 2007) have reported higher response rates in patients with low CA repeats, and longer time to progression (HR 0.54, *P*=0.014; Han *et al*, 2007) and OS (20 *vs* 11 months, RR: 1.89; *P*=0.039; Nie *et al*, 2007). Similarly, an American study (Liu *et al*, 2008) reported improved PFS in patients homozygous for the shorter lengths of CA repeats. This observation was not confirmed by other studies (Gregorc *et al*, 2008), and one study reported an association between shorter CA repeats and poorer survival in the absence of anti-EGFR treatment (Dubey *et al*, 2006). Molecular analysis of the SATURN trial did not confirm predictive value of the number of intron 1 CA repeats (Brugger *et al*, 2009).

In addition to EGFR polymorphisms, much interest is focussed on polymorphisms of the *ABCG2* gene, which codes for a multidrug transporter that has been shown to effectively remove gefitinib and erlotinib from cells (Li *et al*, 2007). The ABCG2 421C>A (Q141K) polymorphism results in a glutamine to lysine substitution in codon 141 and has been associated with increased toxicity in patients treated with gefitinib (Cusatis *et al*, 2006) or with increased concentrations of both gefitinib and erlotinib (Li *et al*, 2007; Rudin *et al*, 2008).

It should be noted that all these data are based on retrospective review of small, single cohort studies, using different definitions of key variables such as ‘short’ or ‘long’ intron 1 CA repeats. Therefore, these studies are unable to properly define the predictive or prognostic role of these polymorphisms in NSCLC patients treated with EGFR TKIs.

No data exist about the role of EGFR polymorphisms as predictors for treatment outcome with anti-EGFR monoclonal antibodies.

## Negative predictive factors

### *EGFR* mutations and resistance to anti-EGFR treatment

One of the mechanisms of primary and acquired resistance in patients who receive TKI treatment is insertion point mutations in exon 20 of the *EGFR* gene. The spectrum of resistant mutations includes the exon 20 insertion mutants D770_N771 (ins NPG), D770_(ins SVQ) and D770_(ins G) N771T (Gazdar, 2009). Nevertheless, it should be noted that these mutations are relatively rare, suggesting that other mechanisms also contribute to primary resistance to EGFR TKI treatment.

Virtually all patients responding to TKI treatment will inevitably develop resistance to these agents. A point mutation in the tyrosine kinase domain (T790M) is found in approximately half of patients at the time of acquired resistance to EGFR TKI therapy (Gazdar, 2009). This mutation has been observed in a small fraction of cells in tumours from pretreated patients, believed to be gained through selective pressure during treatment (Gazdar, 2009). At present, there are insufficient data to treat patients with tumours having classical activating exon 19 or 21 mutations that coexist with exon 20 T790M mutations differently than patients without exon 20 mutations. Physicians should be aware that the detection of resistance mutation may herald the development of clinical resistance to gefitinib or erlotinib.

### K-*RAS*

Ras plays an important role in the EGFR downstream signalling pathway, by activating Raf-kinase, MAPK and promoting cell proliferation (Hynes and Lane, 2005). The K-*RAS* mutations result in EGFR-independent activation of MAPK and are mutually exclusive with *EGFR* mutations (Pao *et al*, 2005). These mutations have been proposed as a mechanism of primary resistance to TKIs in NSCLC and are observed in ∼15–30% of NSCLC patients. Several studies suggest that K-*RAS* mutations are negative predictive factors of response to single-agent TKI treatment in advanced/metastatic NSCLC (Zhu *et al*, 2008). However, the molecular analysis of the SATURN trial showed that the benefit from maintenance erlotinib is similar in patients with and without K-*RAS* mutations in their tumours (HR for PFS 0.77 and 0.70, respectively; Brugger *et al*, 2009). Although several studies support that anti-EGFR monoclonal antibodies are not active in colorectal cancer patients with K-*RAS* gene mutations, it seems that K-*RAS* mutations have no predictive role in NSCLC patients treated with these agents (O’Byrne *et al*, 2009; Khambata-Ford *et al*, 2010), although limited data are available. At present, there are insufficient data to use K-*RAS* mutation status for lung cancer patient selection to EGFR inhibitor therapy.

### Serum proteomic determination of predictive biomarkers for TKIs

Matrix-assisted laser desorption/ionisation, time-of-flight mass spectrometry is a potentially powerful and inexpensive tool for identifying protein signatures in serum. Using this approach, a TKI prediction algorithm was identified using a training set of 139 samples of serum or plasma (Taguchi *et al*, 2007). Based on eight discriminating features and validated in two independent cohorts, it selectively predicted survival in patients who had received an EGFR TKI. In cohort 1, there were 67 patients treated with gefitinib. Survival in the high-risk group was 92 *vs* 207 days in the low-risk group with HR of 0.50 and 95% CIs of 0.24–0.78. In cohort 2, survival was 107 *vs* 306 days with HR of 0.41 and 95% CIs 0.17–0.63. This serum proteomic classifier has been commercially developed (Veristrat) and was shown to associate with outcome in a clinical trial of erlotinib and bevacizumab (Carbone *et al*, 2010a). An 11 proteomic feature-based classifier has been developed that associated with OS in a Cox proportional hazards model in the training set (*P*=0.0006) and also when applied in a blinded test to patients treated with erlotinib alone in the phase II first-line monotherapy trial, ECOG 3503 (*n*=82, *P*<0.0001; Salmon *et al*, 2009). Analysis of the proteomic classifier in the sera from patients included in the BR.21 trial was recently reported and showed that this marker had mainly prognostic role (Carbone *et al*, 2010b).

## Discussion

Identification of predictive markers is important for selection of patients with advanced/metastatic NSCLC who are likely to obtain a clinical benefit from anti-EGFR treatment. A panel of such biomarkers has been extensively evaluated in NSCLC patients treated in clinical trials with these agents. The EGFR expression as determined by IHC should not be considered as a valid predictive marker given that published results are conflicting with some studies showing weak predictive value (mainly placebo-controlled second/third-line trials), not confirmed in other studies. High *EGFR* gene copy number, as assessed by FISH, has been associated with a survival benefit in the placebo-controlled phase III TKI trials (Hirsch *et al*, 2006; Zhu *et al*, 2008), but had no predictive value in randomised trials comparing TKI treatment with chemotherapy (Fukuoka *et al*, 2009; Douillard *et al*, 2010). On the contrary, *EGFR* mutations were associated with a dramatic benefit in terms of PFS in both placebo-controlled (Brugger *et al*, 2009) and chemotherapy-controlled trials (Fukuoka *et al*, 2009; Douillard *et al*, 2010). Furthermore, a recent meta-analysis by Dahabreh *et al* (2010) reported that *EGFR* mutations are predictive of response to TKIs with a higher sensitivity and specificity compared with *EGFR* gene gain, although survival improvement may not be confined exclusively to patients with tumour shrinkage. On the basis of the data from clinical trials comparing EGFR TKIs with chemotherapy, *EGFR-*activating mutation status appears to be the most valid marker for the selection of patients who would derive the most benefit from TKI treatment.

It is not clear why conflicting results are reported between trials. The major strength of the above presented conclusions is that they are based (with the exception of germline EGFR polymorphisms) on data derived from large randomised phase III trials. On the other hand, it should be noted that molecular analyses derived from placebo-controlled studies (BR.21 and ISEL) were retrospective, not preplanned and restricted to patient subsets with available samples and thus likely to be biased (McShane *et al*, 2005). Therefore, all results based on these trials should be considered exploratory (Zhu *et al*, 2008). On the contrary, chemotherapy-controlled trials (INTEREST, IPASS) had a prospective preplanned biomarker analysis. Furthermore, conflicting results about the predictive role of EGFR gene copy number could be explained by possible biological differences between early (first line or maintenance) *vs* late (second and third line) settings. The role of *EGFR* mutations was confirmed in three phase III trials specially designed for the population of patients treated in the first-line setting (Maemondo *et al*, 2010; Mitsudomi *et al*, 2010; Zhou *et al*, 2010).

An important issue is when to use EGFR TKI in patients who have *EGFR* mutations in their tumours – should these agents be administered as first-line, maintenance or as second/third-line treatment? Only comparative data exist to answer this question; no prospective study has been specifically designed to address this issue and cross-study comparisons are not reliable. It is unlikely that a clinical trial will be designed to answer this question, given the large number of patients who will be needed. Given that there is unquestionable benefit in terms of PFS, RR and quality of life in the first-line setting, and that only a subgroup of patients will be suitable for second-line treatment, EGFR TKIs should be recommended in NSCLC patients harbouring *EGFR* mutations for first-line treatment (D’Addario *et al*, 2010).

Another important issue is whether treatment with EGFR TKIs should be denied to patients with tumours showing wild-type *EGFR* gene. The BR.21 study reported that both groups (tumours having *EGFR* mutations or wild-type *EGFR*) derive a survival benefit from treatment with erlotinib compared with placebo, although the effect of erlotinib was much greater in patients with *EGFR-*mutated tumours (Shepherd *et al*, 2005). A similar observation was reported in the SATURN trial (Cappuzzo *et al*, 2010a). Based on the results of this and other trials, EGFR TKI treatment should not be confined to patients harbouring *EGFR* mutations, although the smaller benefit in patients with *EGFR* wild-type tumours should be taken into account in pharmacoeconomic analyses to guide reimbursement decisions. It is likely that a combination of markers, such as K-*RAS* mutations or other as yet unidentified markers, will be used in future to identify patients who will not benefit from EGFR TKI therapy.

An important concern is the feasibility of large-scale screening of NSCLC patients for *EGFR* mutations. A study from the Spanish Lung Cancer Group reported the screening of >2000 NSCLC patients and found mutations in 350 patients (16.6% Rosell *et al*, 2009). Median PFS and OS for 217 patients who received erlotinib were 14 and 27 months, respectively. New techniques of *EGFR* mutation testing, in particular IHC with antibodies constructed against abnormal EGFR proteins as a result of gene mutations, should facilitate large-scale testing (Kitamura *et al*, 2010).

The evolution of our knowledge of biomarkers to guide treatment in NSCLC patients treated with *EGFR* inhibitors came in parallel to the clinical trials testing these agents. This knowledge should serve as a lesson in the current development of other agents in NSCLC, where many trials do not meet their end points because of a questionable clinical benefit in unselected populations. Rational drug development, based on profound understanding of tumour biology, a drug's mechanism of action and clinical implications of patient selection, is hoped to impact on the current poor treatment outcomes of NSCLC patients.

## Figures and Tables

**Table 1:** Survival HRs according to EGFR protein expression in phase III trials with EGFR tyrosine kinase inhibitors

**Trial**	***N***	**HR**	**95**% **CI**	***P***-**value**	**Biomarker** **by** **treatment** **interaction** ***P***-**value**
*BR.21* (Shepherd *et al*, 2005; Tsao *et al*, 2005)					
Positive	184	0.68	0.49–0.95	0.02	NR
Negative	141	0.93	0.63–1.36	0.70	
					
*ISEL* (Thatcher *et al*, 2005; Hirsch *et al*, 2006)					
Positive	264	0.77	0.56–1.08	0.126	0.049
Negative	115	1.57	0.86–2.87	0.140	
					
*SATURN* (Brugger *et al*, 2009; Cappuzzo *et al*, 2010b)^a^					
Positive	NR	0.69	0.58–0.82	<0.0001	NR
					
*ATLAS* (Johnson *et al*, 2009)^a^					
Positive	191	0.92	0.64–1.32	NR	
Negative	67	1.00	0.55–1.82	NR	NR
					
*INTEREST* (Kim *et al*, 2008; Douillard *et al*, 2010)					
Positive	284	1.00	0.77–1.29	0.98	0.87
Negative	96	1.00	0.65–1.55	0.99	
					
*IPASS* (Fukuoka *et al*, 2009; Mok *et al*, 2009)^a^					
Positive	266	0.73	0.55–0.96	0.0243	0.21
Negative	99	0.97	0.64–1.48	0.8932	

Abbreviations: ATLAS=Avastin and Tarceva or Avastin and pLAcebo in patients with NSCLC; CI=confidence interval; EGFR=epidermal growth factor receptor; HR=hazard ratio; ISEL=Iressa Survival Evaluation in Lung Cancer; INTEREST=Iressa NSCLC Trial Evaluating Response and Survival *versus* Taxotere; IPASS=Iressa Pan-Asian Study; NR=not reported; SATURN=Sequential Tarceva in Unresectable NSCLC.

a HR for progression-free survival.

**Table 2:** Survival HRs according to EGFR gene copy number as assessed by FISH in phase III trials with EGFR tyrosine kinase inhibitors

**Trial**	***N***	**HR**	**95% CI**	***P*-value**	**Biomarker by treatment interaction *P*-value**
*BR.21* (Shepherd *et al*, 2005; Tsao *et al*, 2005; Zhu *et al*, 2008)					
FISH positive	61	0.43	0.23–0.78	0.0042	0.12
FISH negative	98	0.80	0.49–1.29	0.3525	
					
*ISEL* (Thatcher *et al*, 2005; Hirsch *et al*, 2006)					
FISH positive	114	0.61	0.36–1.04	0.067	0.045
FISH negative	256	1.16	0.81–1.64	0.417	
					
*SATURN* (Brugger *et al*, 2009; Cappuzzo *et al*, 2010b)^a^					
FISH positive		0.69	NR	0.0001	NR
					
*ATLAS* (Johnson *et al*, 2009)^a^					
FISH positive	87	0.66	0.39–1.13	NR	NR
FISH negative	109	1.40	0.86–2.28	NR	
					
*INTEREST* (Kim *et al*, 2008; Douillard *et al*, 2010)					
FISH positive	174	1.09	0.78–1.51	0.62	0.52
FISH negative	200	0.93	0.68–1.26	0.64	
					
*IPASS* (Fukuoka *et al*, 2009; Mok *et al*, 2009)^a^					
FISH positive	249	0.66	0.50–0.88	0.0050	0.0437
FISH negative	157	1.24	0.87–1.76	0.2368	

Abbreviations: ATLAS=Avastin and Tarceva or Avastin and pLAcebo in patients with NSCLC; CI=confidence interval; EGFR=epidermal growth factor receptor; FISH=fluorescence *in situ* hybridisation; HR=hazard ratio; ISEL=Iressa Survival Evaluation in Lung Cancer; INTEREST=Iressa NSCLC Trial Evaluating Response and Survival *versus* Taxotere; IPASS=Iressa Pan-Asian Study; NR=not reported; SATURN=Sequential Tarceva in Unresectable NSCLC.

a HR for progression-free survival.

**Table 3:** Survival HRs according to EGFR mutation status in phase III clinical trials with EGFR tyrosine kinase inhibitors

**Trial**	***N***	**HR**	**95% CI**	***P*-value**	**Biomarker by treatment interaction *P*-value**
*BR.21* (Shepherd *et al*, 2005; Tsao *et al*, 2005; Zhu *et al*, 2008)					
EGFR mutated	30	0.55	0.25–1.19	0.1217	0.47
EGFR wild-type	176	0.74	0.52–1.05	0.0924	
					
*ISEL* (Thatcher *et al*, 2005; Hirsch *et al*, 2006)					
EGFR mutated	26	NR	NR	NR	NR
EGFR wild-type	189				
					
*SATURN* (Brugger *et al*, 2009; Cappuzzo *et al*, 2010b)^a^					
EGFR mutated	22	0.10	0.04–0.25	<0.0001	
EGFR wild-type	199	0.78	0.63–0.96	0.0195	
					
*ATLAS* (Johnson *et al*, 2009)^a^					
EGFR mutated	52	0.44	0.22–0.86	NR	NR
EGFR wild-type	295	0.85	0.64–1.13	NR	
					
*INTEREST* (Kim *et al*, 2008; Douillard *et al*, 2010)					
EGFR mutated		0.83	0.41–1.67	0.60	0.59
EGFR wild-type	NR	1.02	0.78–1.33	0.91	
					
*IPASS* (Fukuoka *et al*, 2009; Mok *et al*, 2009)^a^					
EGFR mutated	261	0.48	0.36–0.64	<0.001	<0.0001
EGFR wild-type	176	2.85	2.05–3.98	<0.001	
WJTOG3405 (Mitsudomi *et al*, 2010)^a^^,^^b^	177	0.489	0.336–0.710	<0.0001	NA
NEJ002 (Maemondo *et al*, 2010)^a^^,^^c^	230	0.36	0.25–0.51	<0.001	NA
CTONG 0802 (Zhou *et al*, 2010)	154	0.16	0.10–0.26	<0.0001	NA

Abbreviations: ATLAS=Avastin and Tarceva or Avastin and pLAcebo in patients with NSCLC; CI=confidence interval; EGFR=epidermal growth factor receptor; HR=hazard ratio; ISEL=Iressa Survival Evaluation in Lung Cancer; INTEREST=Iressa NSCLC Trial Evaluating Response and Survival *versus* Taxotere; IPASS=Iressa Pan-Asian Study; NR=not reported; NA=not applicable; SATURN=Sequential Tarceva in Unresectable NSCLC; TKI=tyrosine kinase inhibitor.

a HR for progression-free survival.

b Gefitinib *vs* cisplatin/docetaxel.

c Gefitinib *vs* paclitaxel/carboplatin.
